# Apical-out airway organoids as a platform for studying viral infections and screening for antiviral drugs

**DOI:** 10.1038/s41598-022-11700-z

**Published:** 2022-05-10

**Authors:** Georgios Stroulios, Tyler Brown, Giulia Moreni, Douglas Kondro, Alessandro Dei, Allen Eaves, Sharon Louis, Juan Hou, Wing Chang, Dasja Pajkrt, Katja C. Wolthers, Adithya Sridhar, Salvatore Simmini

**Affiliations:** 1 STEMCELL Technologies UK Ltd., Cambridge, UK; 2 STEMCELL Technologies Inc., Vancouver, BC Canada; 3 Department of Medical Microbiology, OrganoVIR Labs, Amsterdam UMC Location University of Amsterdam, Meibergdreef 9, Amsterdam, The Netherlands; 4 Department of Pediatric Infectious Diseases, Emma Children’s Hospital, Amsterdam UMC Location University of Amsterdam, Meibergdreef 9, Amsterdam, The Netherlands; 5 Amsterdam Institute for Infection and Immunity, Infectious Diseases, Amsterdam, The Netherlands; 6 Terry Fox Laboratory, BC Cancer, Vancouver, BC Canada; 7 STEMCELL Technologies China Co. Ltd., Shanghai, China

**Keywords:** Assay systems, Stem-cell biotechnology, Biotechnology, Cell biology, Drug discovery, Stem cells

## Abstract

Airway organoids are polarized 3D epithelial structures that recapitulate the organization and many of the key functions of the in vivo tissue. They present an attractive model that can overcome some of the limitations of traditional 2D and Air–Liquid Interface (ALI) models, yet the limited accessibility of the organoids’ apical side has hindered their applications in studies focusing on host–pathogen interactions. Here, we describe a scalable, fast and efficient way to generate airway organoids with the apical side externally exposed. These apical-out airway organoids are generated in an Extracellular Matrix (ECM)-free environment from 2D-expanded bronchial epithelial cells and differentiated in suspension to develop uniformly-sized organoid cultures with robust ciliogenesis. Differentiated apical-out airway organoids are susceptible to infection with common respiratory viruses and show varying responses upon treatment with antivirals. In addition to the ease of apical accessibility, these apical-out airway organoids offer an alternative in vitro model to study host–pathogen interactions in higher throughput than the traditional air–liquid interface model.

## Introduction

The proximal airway epithelium plays a vital role in respiration by allowing air flow to and from the distal lungs and acting as the first line of defence against airborne pathogens. As such, the generation of relevant in vitro airway models has become a significant research focus. The air–liquid interface (ALI) culture system has proven to be paramount in this endeavour as it allows for easy access to both the apical cell surface, which is exposed to the luminal external environment in vivo, and the basolateral surface. Moreover, it contains all the major airway epithelial cell types such as ciliated cells, mucus producing goblet cells, club cells, and basal cells, with the latter functioning as the regional stem cells of the airway epithelium^1^. Establishment of an ALI culture system relies heavily on the use of a tissue culture plate insert, which is composed of a permeable membrane and has limited scalability. To overcome this limitation, alternative culture systems such as organoids and spheroids, have been developed. Organoids are generated embedded in an extracellular matrix (ECM), typically Matrigel, which raises standardization issues such as the poorly-defined composition of Matrigel^2^ and the introduction of nutrient gradients^3^. Nonetheless, since airway organoids resemble the cellular composition of the human airway epithelium^4,5^, they have been employed to model host–pathogen interactions^6,7^. These organoids are characterized by a polarized apicobasal epithelium encircling a central lumen that exposes the basal membrane to the medium^6^ and limits access to the apical surface of the epithelium, making the study of host–pathogen interactions challenging.

This basal-out polarity is inherent in all epithelial organoid culture systems. Therefore, a variety of methods have been developed to allow access to the apical membrane^7^, with some of these techniques being successfully adapted into airway organoid cultures. For instance, mechanical fragmentation of airway organoids exposes the apical surface to the medium, and has been used to study the interactions of Respiratory Syncytial Virus^5^, Enterovirus A71^8^ and Influenza^6,9,10^. However, this method does not allow for the accurate modelling of the infection since both the apical and basal surfaces of the organoid epithelium are exposed to the pathogen, potentially eliciting non-specific responses. An alternative approach uses microinjections to deliver pathogens directly to the organoid’s lumen, thereby preserving its 3D structure. This method has been instrumental in studying the effects of *Cryptosporidium* on airway organoids^11^ but it is time-consuming and has limited potential for high-throughput applications.

A possible solution has been described by Co et al. who demonstrated that ECM-embedded enteric organoids can reverse their polarity when they are transferred into an ECM-free suspension culture^12^. This method was applied to proximal airway organoid cultures, generating differentiated organoids with inverted polarity that displayed cilia on the outer side^13^. However, the inversion of the polarity requires complete removal of the ECM proteins whilst maintaining the organoids intact. Moreover, culturing ECM-derived organoids in suspension often leads to organoid-organoid fusion events^14^, which severely impacts organoid output. Recently, Boekig et al. described the generation of a mixed population of ECM-embedded airway organoids, the majority of which presented their apical membranes externally. These organoids were generated by embedding in a mixture of PureCol and Matrigel, indicating that modifying ECM conditions can influence organoid polarization^15^. Others have also described the spontaneous generation of airway spheroids with outward facing cilia from epithelial sheets or single cell suspensions from tissue explants^16–19^.

Organoids have great potential for high-throughput applications, but the lack of standardization hinders their inclusion, with the relatively long differentiation times and the reliance on ECMs further aggravating the situation. Here, we describe a novel ECM-free method to generate apical-out airway organoids (Ap-O AO) by differentiating aggregates derived from 2D-expanded human bronchial epithelial cells (hBECs). The complete absence of ECM and the use of micropatterned plates followed by suspension culture, can overcome the obstacles that ECM-embedded airway organoids face, and may allow the utilization of Ap-O AO in high-throughput assays such as antiviral drug screenings. For this purpose, we show infectivity of the Ap-O AO by respiratory viruses as well as inhibition of viral replication by antivirals.

## Results

### Establishment of an ECM-free method to generate Ap-O AO

**Figure 1:**
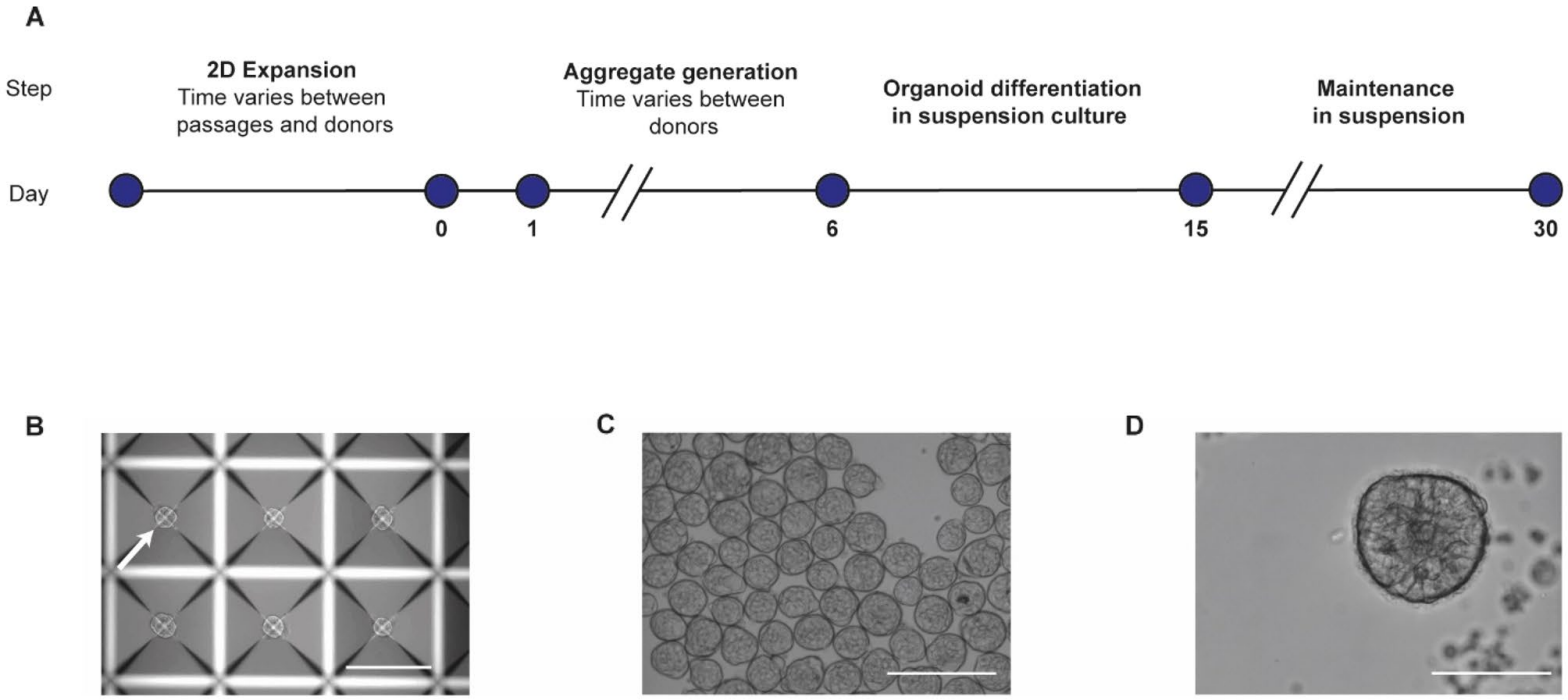
Generation of airway organoids with apical-out orientation from hBEC aggregates. (**A**) Workflow for the generation of apical-out organoids showing the time requirement for each step. (**B**) Representative brightfield image of aggregates generated by hBECs after 1 to 6 days of incubation in a microwell. Arrow points at a representative aggregate located in the centre of the microwell (scale bar 300 μm). (**C**) Representative brightfield image of aggregates after initiation of suspension culture (scale bar 200 μm). (**D**) Representative brightfield image of terminally differentiated Ap-O AO at day 15. Cilia can be detected at the edge of the organoid (scale bar 100 μm).

The presence of ECM during organoid formation guides the polarity of the resulting organoids, directing the apical side of the epithelium toward the central lumen^12^. Therefore, we examined whether we could generate aggregates of 2D expanded hBECs in an ECM-free environment and differentiate them towards Ap-O AO in suspension culture (Fig. 1A). We leveraged the potential of the AggreWell 400 platform of micropatterned plates to promote the generation of a homogenous-sized population of aggregates in the absence of ECM proteins^20,21^. To optimize the seeding density that would allow the efficient generation of Ap-O AO, we expanded hBECs in 2D culture in PneumaCult-Ex Plus Medium and seeded between 100 and 500 hBECs in each microwell (Supplementary Fig. 1A) of an AggreWell plate. After incubating one to 6 days, all tested seeding densities were able to give rise to numerous aggregates (Fig. 1B). To induce the differentiation of the aggregates towards organoids, we resuspended the aggregates in the same culture media and transferred them into a new tissue culture plate pre-treated with Anti-Adherence Rinsing Solution to prevent adhesion to the plastic substrate (Fig. 1C, Supplementary Fig. 1A). This ECM-free suspension culture condition allows for easy monitoring of differences in dimensions of the aggregates generated in the tested seeding densities. Analysis of the size of the aggregates confirmed the positive correlation between the initial number of hBECs seeded in microwells and the size of the aggregates transferred in suspension between days 1 and 6 (Supplementary Fig. 1B). Maintaining the organoids in the same medium would streamline the process, therefore we tested if PneumaCult Apical-Out Airway Organoid Medium could support not only the aggregation of hBECs in micropatterned plates, but also their differentiation towards Ap-O AO. Aggregates cultured in static suspension conditions display ciliogenesis by day 10, and by day 15 we detected organoids composed of a cell-dense core and an epithelium that present ciliated cells on the outer side, suggesting an apical-out polarity (Fig. 1D). These cilia demonstrated a coordinated beating movement, which propelled the organoids in suspension (Supplementary Video 1). However, during organoid differentiation, we observed cells being shed from all the tested seeding density conditions, with larger aggregates shedding more cells than smaller aggregates. This shedding resulted in all Ap-O AO having the same diameter of approximately 104.2 ± 28.2 μm by day 15 (Supplementary Fig. 1B). Since different seeding densities gave rise to Ap-O AO of similar size, to maximize efficiency we decided to aggregate 100 cells per microwell, as it was the density with the lowest size distribution range. These results indicate for the first time that functional epithelial terminally differentiated organoids, such as airway organoids, can be generated with an apical out configuration in an ECM-free environment by differentiating aggregates in suspension culture.

### Ap-O AO can be generated efficiently across multiple passages and demonstrated robust ciliogenesis

**Figure 2:**
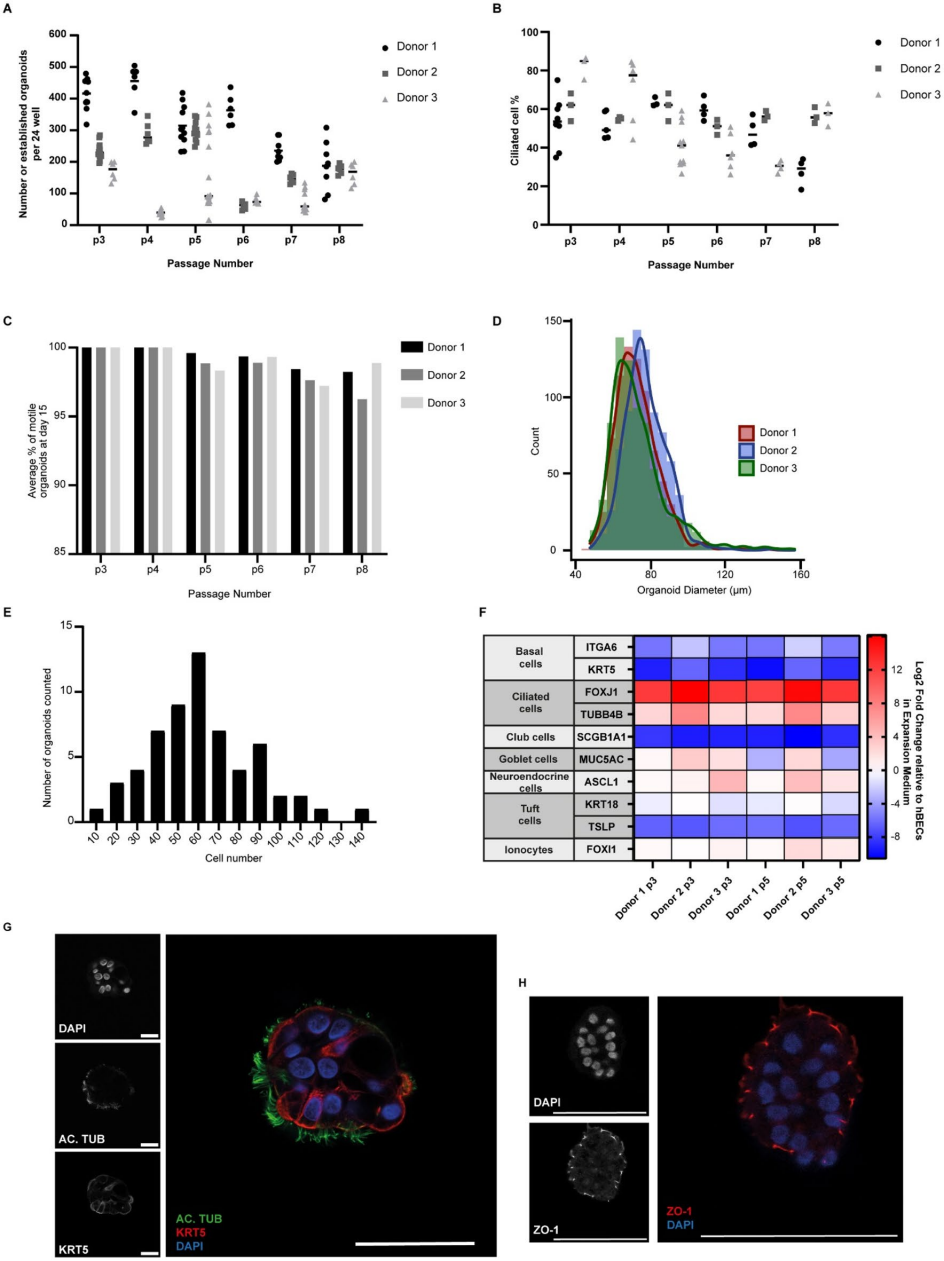
Characterisation of apical-out airway organoids. (**A**) Number of generated organoids per well of a 24-well tissue culture plate plate across three different donors and multiple passages. Data points represent independent wells of a 24-well plate (n = 3). (**B**) Ciliated cell percentage of generated apical out organoids at day 15 across multiple passages. Data points represent measurements taken from independent wells of a 24-well plate (n = 3). (**C**) Percentage of organoids counted for panel (**A**) that displayed motile cilia on the outside at day 15 across three different donors and multiple passages. Organoids were classified as ‘motile’ when displaying sufficient cilia to cause motion of the organoid. (**D**) Histogram depicting the frequency of different Feret diameters in p3 Ap-O AO from 3 donors. 750 organoids were equally counted from 3 wells for each donor. (**E**) Histogram depicting the frequency of measured DAPI-positive cells in each organoid. In total 60 p3 organoids were counted. (**F**) Fold changes in the RNA levels of common differentiation markers in Ap-O AO across 2 different passages in three donors. Results were normalized against p3 hBECs cultured in PneumaCult-EX Plus (n = 3). (**G**) Expression of AC. TUB (green) and KRT5 (red) in Ap-O AO. Nuclei are visualized with DAPI (blue). Single channels are depicted in grayscale (scale bars = 50 μm). (**H**) Expression of ZO-1 (red) in Ap-O AO. Nuclei are visualized with DAPI (blue). Single channels are depicted in grayscale (scale bars = 50 μm).

After establishing a new workflow to generate Ap-O AO, we validated the method by examining the organoid formation efficiency across a number of culture passages (p) and donors. To do this, we generated Ap-O AO by seeding 100 2D-expanded cells derived from 3 donors from passages 3 to 8 in microwells and measuring the number of generated Ap-O AO at day 15. Overall, the data demonstrated variability (minimum = 15, maximum = 504 per 24-well of a tissue culture plate) in the number of apical out organoids obtained from different passages and donors, although all donors were capable of generating approximately 400 organoids on average per 24-well AggreWell well (Fig. 2A). We then assessed the differentiation potential of the hBECs in our ECM-free workflow by counting the percentage of ciliated cells within organoids at different passages. Ciliated cells were present in organoids for a minimum of 8 passages, but their proportion could fluctuate at any given passage (Fig. 2B). We also quantified the number of organoids that are motile in suspension and observed a high degree of homogeneity in the cultures, as nearly all the organoids displayed beating cilia on the outer side, indicating their successful apical-out orientation (Fig. 2C). The homogeneity of the culture is further pronounced by the size of generated organoids (Fig. 2D), which was found to be consistent between all donors tested (average diameter of 72.6 ± 6.7 μm, 77.3 ± 4.7 μm and 73.6 ± 0.3 μm for donors 1, 2 and 3 respectively with coefficients of variance of 14.2 ± 1.5%, 13.6 ± 1.1% and 21.0 ± 1.1% respectively). In order to verify the organoids’ homogeneity at the cellular level, we quantified the number of cells that Ap-O AO were composed of. Passage 3 Ap-O AO were fixed, stained and imaged for DAPI to visualize and count the number of nuclei. Ap-O AO on average were composed of 61.8 cells with a 95% confidence interval of 6.77 (Fig. 2E). These results indicate that hBECs maintain a differentiation potential when generating Ap-O AO and can be used in downstream applications even in later passages, in contrast to ALI cultures where a loss of their pseudostratified morphology is observed as early as passage 4^22^.

Recently, other studies using intestinal^12,14^ and pulmonary organoids^13^ have demonstrated that the polarity of the basal-out organoids maintained in Matrigel could be reversed by removing the ECM from the cultures. To compare the two approaches, we measured the efficiency of Ap-O AO formation of our ECM-free workflow and the previously published method of ECM removal. We observed that the ECM-free workflow consistently generates more Ap-O AO across all donors tested, with at least a five-fold increase in efficiency (Supplementary Fig. 2). Not all donors tested were successful in generating Ap-O AO using the ECM-removal method since the quality of those cultures rapidly deteriorated when in suspension phase (Supplementary Fig. 3A). In addition, organoid fusion is commonly observed in suspension culture 15 days after ECM removal (Supplementary Fig. 3B).

To further characterise the Ap-O AO, we collected terminally differentiated Ap-O AO at day 15 and performed quantitative PCR analysis to assess the levels of common airway epithelial cell markers (Fig. 2F). When compared against the levels of 2D-expanded hBECs at passage 3, the abundance in terminally differentiated Ap-O AO of basal cell markers^1^ *ITGA6* and *KRT5* was reduced. In contrast, the expression of ciliated cell markers *FOXJ1,* a transcription factor involved in cilia production, and *beta-TUBULIN IV* (TUBB4B), a component of the cilia axoneme, was upregulated*.* The relative levels of cell markers from the secretory lineage, such as *SCGB1A1* (club cells)^23^ and *MUC5AC* (goblet cells) did not reveal the presence of these cell types in Ap-O AO. The expression of other markers for rather rare cell types such as Tuft cells (*KRT18*, *TSLP*)^24,25^, neuroendocrine cells (*ASCL1*)^25^ and ionocytes (*FOXI1*)^25^ could not be detected. These changes were consistent across all tested passages and donors.

In order to validate the mRNA expression profiles, we immunostained terminally differentiated Ap-O AO at day 15 to detect ciliated, goblet and basal lineage markers. Our results demonstrate that Ap-O AO contain ciliated cells, as confirmed by the presence of acetylated TUBULIN (AC. TUB) on the outward-facing apical cell surface (Fig. 2G). KERATIN5-expressing basal cells were present alongside ciliated cells (Fig. 2G), but MUC5AC was not detected, indicating that the Ap-O AO did not contain goblet cells. Cell–cell tight junctions could be readily visualized, since ZO-1 was highly expressed (Fig. 2H). These results indicate that our method supports the efficient generation of differentiated Ap-O AO across multiple passages and donors.

As this system has the potential to generate large numbers of Ap-O AO, we decided to test its scalability using the 6-well plate format of the AggreWell 400 to increase organoid production. In total, the number of organoids obtained per well of a 6-well AggreWell 400 plate was found to be on average 2316 ± 368 organoids (Supplementary Fig. 4A). This is approximately 3 times higher than the number of Ap-O AO obtained per well in the 24-well plate format, which reflects the difference in microwell content of the used platforms. These organoids demonstrated similar differentiation potential (Supplementary Fig. 4B) and morphology (Supplementary Fig. 4C) to those generated from 24-well AggreWell plates (Figs. 1D, 2B). Furthermore, use of the 6-well AggreWell plate format appeared to robustly preserve the high degree of size homogeneity observed in Ap-O AO generated in 24-well AggreWell plate (Fig. 2D), as the organoids had an average diameter of 93.5 μm and an interquartile range of 25.99 μm (Supplementary Fig. 4D). Ap-O AO generated in parallel in a 24-well AggreWell plate had a similar average Feret diameter of 86.6 μm and an interquartile range of 21.08 μm. However, aggregate-aggregate fusion was observed more frequently in organoids generated using the 6-well AggreWell platform than the 24-well, resulting in a wider distribution of the measured organoids. These results strongly suggest that Ap-O AO is a culture system that is suitable to scale-up production to potentially support high throughput downstream applications.

### Ap-O AO are susceptible to infection by common respiratory viruses

**Figure 3:**
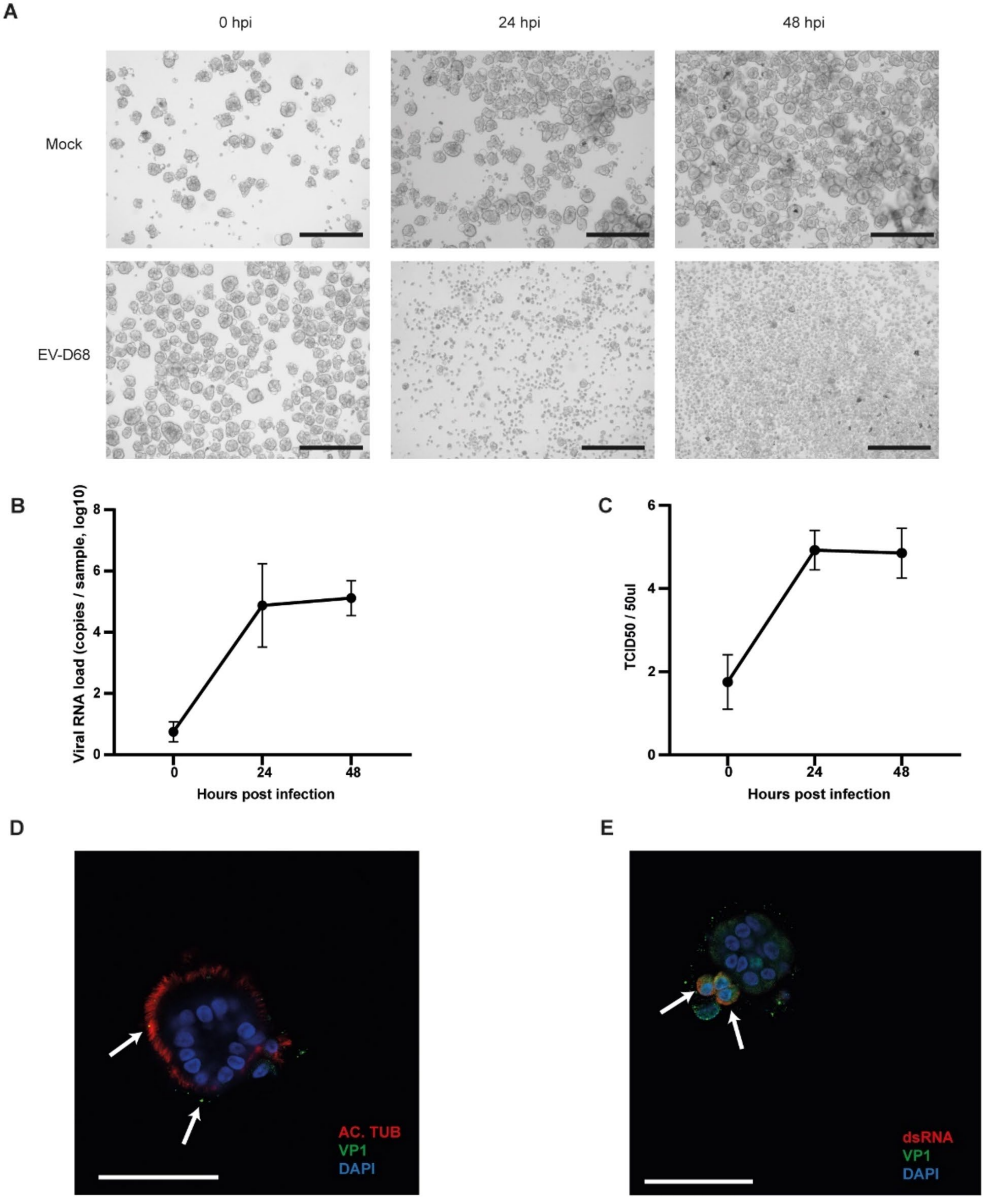
Ap-O AO are susceptible to infection with EV-D68. (**A**) Representative brightfield images showing the cytopathologic effect of EV-D68 infection over 48 h. Infected Ap-O AO organoids disintegrate to single cells over the course of the infection (scale bars = 300 μm). (**B**) Viral load of EV-D68 detected by qPCR in the supernatant. Points represent mean values of all biological replicates. Error bars represent the SD (n = 3). (**C**) Viral titer detected in the sampled medium at 0 to 48 h post infection. Titers are expressed as TCID_50_. Points represent mean values of all biological replicates. Error bars represent the SD (n = 3). (**D**) Distribution of AC. TUB (red) and the bound viral particles with VP1 (green) in Ap-O AO 0 hpi with EV-D68. DAPI (blue) visualises the nuclei. Arrows indicate bound viral particles (scale bar = 50 μm). (**E**) Apical-out airway organoid 6 hpi with EV-D68. The nuclei are visualized with DAPI (blue), VP1 (green) and ds-RNA (red) indicate active viral replication. Arrows point at infected cells with ongoing replication (scale bars = 50 μm).

Viral infections of the respiratory tract are the most common illnesses worldwide^26^. It is therefore crucial to be able to model them in vitro, as well as to identify new potential treatments. To determine if the AP-O AO system is applicable for respiratory virus research, we exposed the organoids to several common respiratory viruses causing human disease. EV-D68 is an emerging respiratory pathogen that replicates well on ALI cultures infecting both ciliated and non-ciliated cells^27^. To assess whether our Ap-O AO were susceptible to infection with EV-D68, we infected day 15 differentiated organoids with EV-D68. Twenty-four hours post-infection, the cytopathogenic effect (CPE) of the viral infection was evident, with only a few intact organoids detected (Fig. 3A bottom middle panel), and at 48 hpi all the organoids were dissociated to single cells (Fig. 3A bottom right panel). However, not all donors displayed the same levels of CPE (Supplementary Fig. 5), indicating that inter-donor variability was present and could be detected in this system. Mock infected organoids maintained a healthy morphology and did not exhibit any CPE over the duration of the incubation (Fig. 3A, top panels).

Since the viral progeny are shed from the apical side, the apical-out polarisation allows for the easy monitoring of the viral titers during the course of the infection. To do so, media samples were taken at designated time points to assess viral RNA quantity. We quantified the residual viral loads remaining after the final wash step of the inoculation and the subsequent viral replication in 24 h intervals. An increase in the viral RNA was detected at 24 hpi by qPCR, indicating successful viral infection and replication (Fig. 3B). In order to evaluate if the detected shed virus was infectious, we assessed the 50% tissue culture infective dose (TCID50) at the same time points. Infectious viral titers generated from Ap-O AO followed a similar pattern to the detected RNA load, indicating the generation of new infectious viral particles (Fig. 3C).

To confirm that EV-D68 effectively infected the organoids, we fixed Ap-O AO after infection with EV-D68 and performed immunostaining of the capsid protein VP1. Viral particles were detected both intra- and extracellularly in infected Ap-O AO (Fig. 3D,E, Supplementary Fig. 6A). As the infection progresses, the viral genome uses a double-strand (ds-) RNA form to generate multiple copies^28^. Cells containing ds-RNA, indicating an ongoing viral replication cycle, were readily observed in Ap-O AO 6 hpi (Fig. 3E, Supplementary Fig. 6B). The absence of the viral signal in non-infected organoids further confirmed the specificity of the stain (Supplementary Fig. 6C).

**Figure 4:**
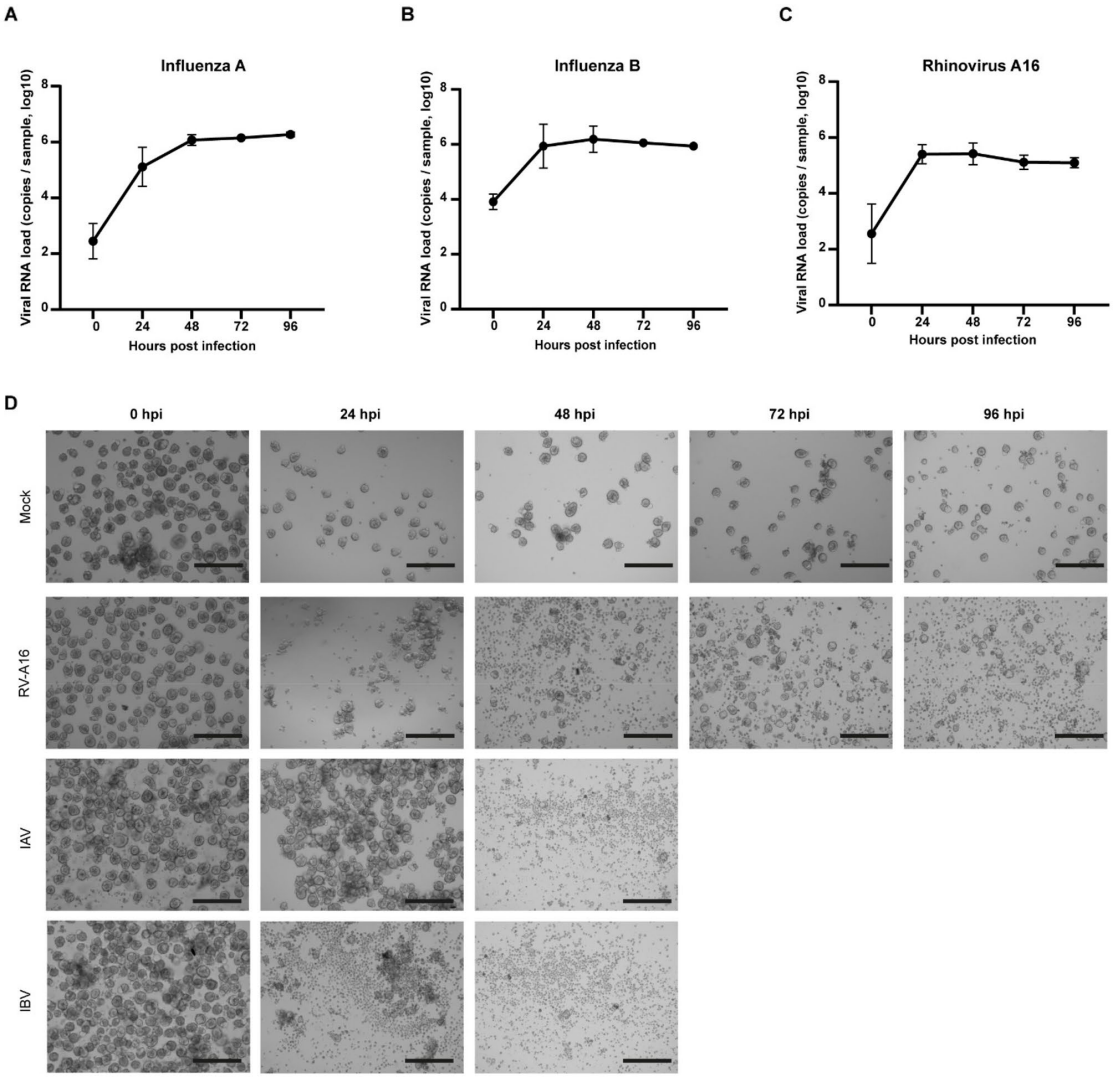
Ap-O AO are susceptible to infection with RV-A16, IAV and IBV. Viral load of IAV (**A**), IBV (**B**) and RV-A16 (**C**) detected by qPCR in the supernatant. Apical-out airway organoids show susceptibility to infection with the tested viruses, and can support the generation of high viral titers. Points represent mean values of all biological replicates. Error bars represent the SD (n = 3). (**D**) CPE following infection with RV-A16, IAV and IBV. Infected Ap-O AO tend to disintegrate to single cells over the course of the infection with the two Influenza viruses. Single organoids can still be observed at 96 hpi with RV-A16 (scale bars = 300 μm).

To further elucidate the applicability of the Ap-O AO in viral research, we infected terminally differentiated (day 15) Ap-O AO derived from 3 different donors with IAV, IBV and RV-A16 and assessed their susceptibility (Fig. 4). Media samples were harvested at 24 h intervals and used to monitor and quantify the viral RNA load of IAV (Fig. 4A), IBV (Fig. 4B) and RV-A16 (Fig. 4C). A marked increase in the RNA load was observed in all viral infections during the first 24 h and remained relatively stable following that. CPE could be observed in infected Ap-O AO across all 3 tested viruses (Fig. 4D). While in the Mock condition (Fig. 4D, first row) organoids remained intact across all the tested time points, shedding of cells could be detected from Ap-O AO infected with RV-A16 as early as 24 hpi (Fig. 4D, second row). However, RV-A16 seemed to have a mild CPE on the Ap-O AO as intact organoids could still be observed as late as 96 hpi. IAV (Fig. 4D, third row) had minimal CPE during the first 24 hpi whereas after 48 hpi, all Ap-O AO disintegrated into single cells. Infection with IBV had a similar progress (Fig. 4D fourth row) to that of IAV, as all the organoids disintegrated after 48 hpi. The major difference between IAV and IBV was observed at 24 hpi, where shed single cells could be detected around the organoids infected with IBV. Here we report that Ap-O AO are susceptible to infection using a panel of different viruses targeting the human airways, supporting the possible application of Ap-O AO for viral assays.

### Ap-O AO to assess antiviral effects

**Figure 5:**
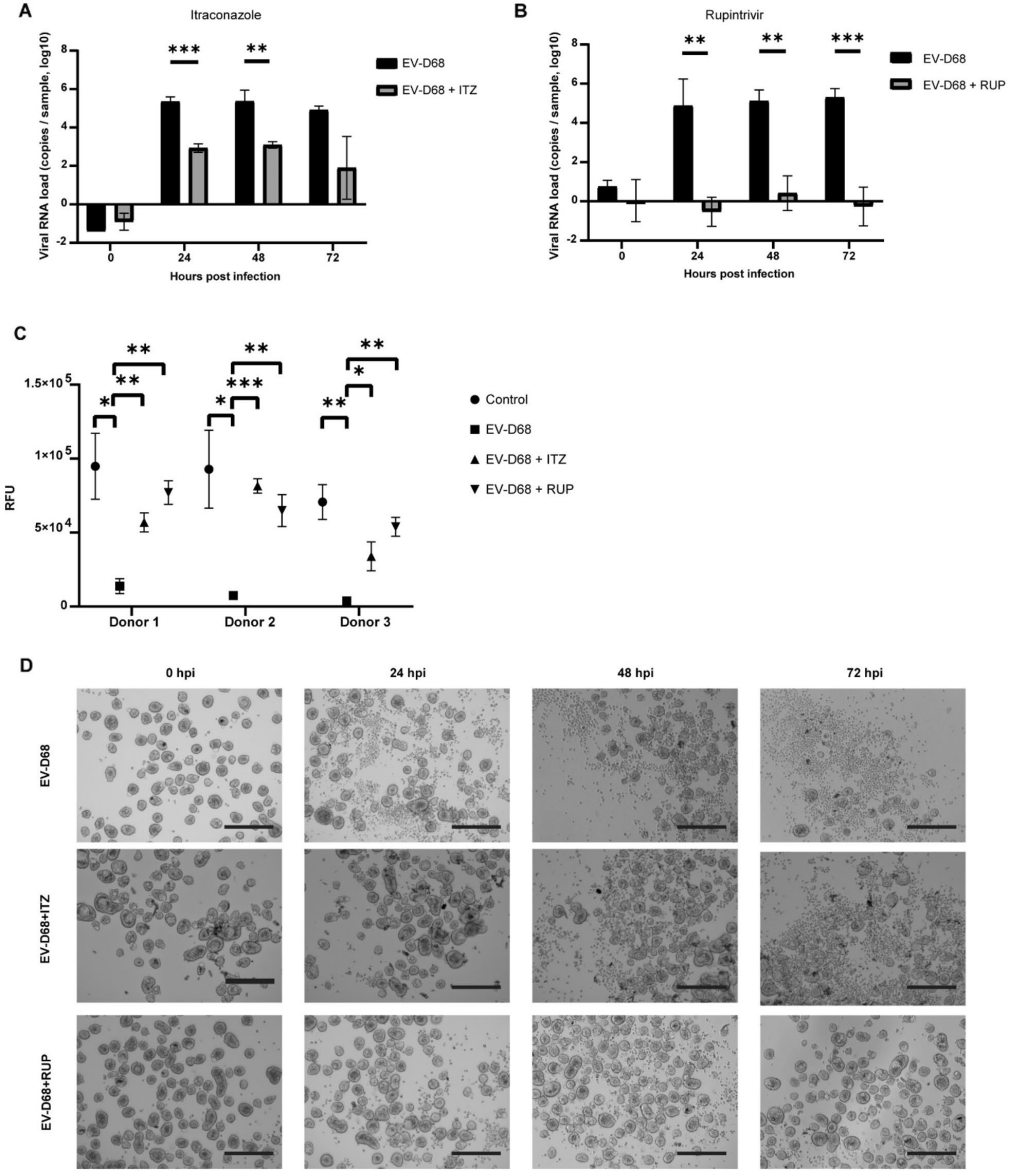
Ap-O AO can be utilised to study antiviral effects on EV-D68 infection. (**A**) Viral load of EV-D68 detected by qPCR in the supernatant in the presence and absence of ITZ. Points represent mean values. Error bars represent the SD. Double asterisks indicate *p* < 0.01 and three asterisks *p* < 0.001. (n = 3). (**B**) Viral load of EV-D68 detected by qPCR in the supernatant in the presence and absence of RUP. Points represent mean values. Error bars represent the SD. Double asterisks indicate *p* < 0.01 and three asterisks *p* < 0.001. (n = 3). (**C**) Relative Fluorescent Units (RFU) measured at 72 hpi indicating changes in viability of Ap-O AO following infection with EV-D68 in the presence or absence of ITZ and RUP. Single points represent the mean with SEM. Single asterisks indicate *p* < 0.05, double asterisks *p* < 0.01 and three asterisks *p* < 0.001. (n = 3). (**D**) Representative images (Donor 2) showcasing the CPE observed in Ap-O AO following infection with EV-D68 without antiviral treatment (top row) and with addition of ITZ (middle row) or RUP (bottom row). (scale bars = 300 μm).

Apical-out airway organoids have the potential to evaluate the neutralizing or inhibiting effect of antiviral drugs, as they offer a relevant platform that can be easily scaled up to accommodate high-throughput applications. Rupintrivir (RUP) is an irreversible inhibitor of Rhinovirus 3C proteases with broad antiviral effects, which was shown to be able to inhibit the replication of EV-D68 in ALI cultures^29^. Itraconazole (ITZ) is a known antifungal drug that inhibits CYP51, a host enzyme required for sterol biosynthesis^30^. ITZ has been shown to reduce the replication of Enterovirus A71^31^ and it may have possible inhibitory effects on other picornaviruses, such as EV-D68. To test if Ap-O AO could be utilized in antiviral drug discovery, we infected Ap-O AO with EV-D68 and assessed the inhibiting effects of these antivirals on viral titers and CPE (Fig. 5). Day 15 differentiated Ap-O AO were infected with 0.05 MOI of EV-D68 and incubated in the presence or absence of 2.5 µM RUP or 5 µM Itraconazole. The infected untreated organoids (EV-D68) displayed high viral RNA titers (Fig. 5A,B) and CPE (Fig. 5D, top row), as was previously described. EV-D68 caused a significant decline of the organoids’ viability in all donors (EV-D68) when compared to the mock-infected organoids treated with the vehicle alone (Control) (Fig. 5C). The addition of the vehicle and the two drugs (Control + ITZ and Control + RUP) to the culture medium does not appear to have a significant detrimental effect on the organoids’ viability compared to the sample with (Control) and without (Control -veh) the addition of the vehicle (Supplementary Fig. 7A). The morphology of the drug-treated organoids (Control + ITZ, Control + RUP) seemed also to be comparable to the untreated ones (Control) (Supplementary Fig. 7B). ITZ significantly inhibited the replication of EV-D68, resulting in approximately 2 orders of magnitude reduction of EV-D68 RNA titers (EV-D68 + ITZ) (Fig. 5A) and significantly increasing the viability of the infected organoids (Fig. 5C). The inhibitory effect of ITZ was also observed in the levels of CPE, as intact organoids could be identified even at 72 hpi in all donors (Fig. 5D middle panel). Similarly, RUP (EV-D68 + RUP) could effectively and significantly inhibit EV-D68 production (Fig. 5B). The ATP levels, indicating the overall viability of the culture, were significantly higher in samples treated with RUP and comparable to the control (Fig. 5C). The inhibitory effect of RUP was further confirmed by the absence of CPE in infected organoids when treated with the drug (Fig. 5D, bottom panel). Our results indicate that infected Ap-O AO are suitable for antiviral drug screening and therefore have potential to be used in high-throughput drug screening applications.

## Discussion

In this work, we describe a novel workflow for the generation of airway organoids that expose their apical side to the culture medium. This method benefits from the robust proliferation of hBECs in 2D, as well as their ability to aggregate and generate large numbers of Ap-O AO. These Ap-O AO developed prominent cell–cell junctions and were composed of basal cells, as well as differentiated ciliated cells. Ciliated cells displayed functional characteristics similar to those observed in vivo, with cilia moving in a cohesive manner. Furthermore, Ap-O AO were shown to be susceptible to infection with Enterovirus D68, Influenza A, Influenza B and Rhinovirus A16 and applicable to antiviral drug screening assays.

The ALI culture system has been the main model for studying airway-specific host–pathogen interactions. Easy access to both the apical and the basal side, as well as the cellular composition that closely resembles the tissue in vivo, have contributed to the understanding of the early stages of pathogen-related lung diseases. Yet, the relatively low scalability of this culture system hampers its utilization in high-throughput applications. To overcome this limitation, spheroids comprised of differentiated airway cells exposing their apical side have been generated from airway epithelial fragments cultured in suspension^16^. Even if this method benefited from the absence of ECM hydrogels, it still displayed low scalability potential due to the direct use of biopsies and lack of a cellular expansion stage. Using a similar approach, Guimbellot et al., successfully generated nasal epithelial spheroids derived from expanded primary cells^19^. Yet, the standardisation of both these methods is hampered by the use of bovine serum and pituitary extract respectively, as well as the differences of the sheet size that resulted in a heterogeneous nasospheroid population with pronounced size heterogeneity.

Other studies were successful in generating spheroids from tissues when submitted in continuous horizontal rotation directly after nasal polypectomy^18^ or from fetal airway derived single cells^17^ in the presence of semi-defined serum substrates. However, the efficiency of the approach is not documented and the differentiation of cells directly after isolation limits utilisation similarly to the aforementioned approaches.

Sporadic generation of airway organoids exposing their apical side to the environment, while still embedded in ECM, has been previously described^6^. Dilution of Matrigel with PureCol resulted in a heterogeneous population of organoids, where approximately 60% presented an apical-out configuration with cilia detected on the outer side. This approach benefited from a modular, non-proprietary medium; however, use of the undefined bovine pituitary extract and Matrigel might cause batch-to-batch inconsistencies and limit standardisation^15^.

Absolute removal of the surrounding ECM from the organoid and the subsequent culture in suspension has been previously shown to induce inversion of the organoid’s polarity^12–14^. Although this method allowed easy access to the apical side of the organoid epithelium, it proved to be rather inefficient, as only the absolute removal of the ECM proteins resulted in effective inversion of the polarity. However, the main limitation of this procedure is the frequent organoid-organoid fusion observed when ECM is removed^32^, which severely impacts the organoid output^14^. As the initial generation of the organoids relies on ECM hydrogels, the induced polarity inversion protocol is affected by morphogen gradients that are established due to diffusion limitations^3^. The high variability of phenotypes induced by these gradients reduces the standardisation of this assay, further hampering its inclusion in high-throughput applications. When applied to airway organoids, this method resulted in the generation of apical out airway organoids composed not only of ciliated and basal cells, but also of club cells. Yet, when compared to ALI cultures, these organoids showed a different cell tropism following infection with SARS-CoV-2^13^.

The distinct characteristics displayed by the Ap-O AO described in this study could help overcome most of the limitations that current airway models are hampered with. Similar to ALI cultures, Ap-O AO readily expose their apical surface and are susceptible to infection with a number of different viruses. The shedding of viral progeny from the apical surface allows easy monitoring of the infection process by simple media sampling. These are in contrast with ECM-embedded apical-in organoids where the apical side is not accessible, which severely limits not only initial infection but also viral titer assessment if the organoid’s structural integrity needs to be preserved. The effects induced by viral infection and antiviral drug treatments were apparent in the viability of the organoids. Moreover, they can be easily monitored by observation of the CPE, which could potentially prevent the requirement of extra lengthy and costly downstream analysis. Our ECM-free system also allows for a fast readout of the cilia beating, indicating that Ap-O AO could potentially be used to model ciliary dyskinesia, or other ciliopathies that are characterized by cilia movement alterations. Ultimately the absence of ECM removes the spatial limitation imposed by the use of ECM domes in standard culture conditions. In addition to this, our method demonstrates that the aggregation of a critical mass of cells, in addition to the specific medium that is being applied, is sufficient to drive the generation of ECM-free Ap-O AO in only 15 days. Aggregates formed by a higher number of cells than required resulted in the shedding of excess cells into the medium, which could be easily removed by performing media changes. Contrary, in ECM-embedded apical-in organoids dead cells are commonly shed into the central lumen, where they are not cleared and can potentially exert damaging or other effects by influencing paracrine signaling^33^.

The use of either 24 or 6-well AggreWell plates for the generation of Ap-O AO showcases that the production is now only limited by the number of microwells used to generate the initial population of hBECs aggregates. Utilisation of the 6-well AggreWell plates generated a much larger population of Ap-O AO than the 24-well plate, with similar characteristics. However, Ap-O AO generated from the 6-well AggreWell plate appeared to have a wider size distribution, potentially due to increased aggregate-aggregate fusion. In addition to this, utilisation of the 6-well plate might pose technical challenges that are not present in the 24-well plate format, such as performing media changes without disrupting the forming aggregates from the microwells. Optimisation of this plate format has the potential to further improve the efficiency of the protocol. Overall, this culture system is the first one that can effectively overcome the scalability limitation, which in addition to the absence of ECM and the highly homogeneous organoid phenotype could introduce a high level of standardization and make Ap-O AO an enticing model for higher-throughput assays. As Ap-O AO could model the effect of antivirals following EV-D68 infection, they have the potential to be utilised in antiviral drug-screening assays. The high degree of homogeneity would also allow for a drastic reduction of the amount of time required to plate the required conditions, as the infection for each technical replicate could be performed in one tube and then the appropriate amount of organoids would be dispersed for each condition to be tested. However, the cellular composition of Ap-O AO might limit their application to assays requiring the presence of basal or ciliated cells only. The absence of cells from the secretory lineage in terminally-differentiated organoids hinders their utilisations in studies focusing on airway epithelial characteristics such as mucus production and cell–cell interactions beyond those observed between basal and ciliated cells.

In conclusion, we have generated an ECM-free workflow that allows the efficient generation of airway organoids that expose their apical side to the media. These organoids are susceptible to infection with a variety of viruses targeting the human airway epithelium and can be utilized to assess the effect of antivirals in virus replication. The absence of an animal-derived ECM scaffold, the high efficiency and the relative short duration of the assay, offers the opportunity to scale up this method in a cost-effective manner and make it suitable for ultra-high-throughput downstream applications in the context of both infectious and other disease modelling.

## Materials and methods

### Primary human airway epithelial cells

Primary hBECs used in the study are from non-smoking, healthy donors and commercially available from either LONZA (cat # CC-2540S) or EPITHELIX SARL (cat # EP51AB).

### hBEC expansion

Initial cryopreserved hBECs were seeded as passage one into T25 cell culture tissue flasks with PneumaCult-Ex Plus Medium (STEMCELL Technologies, catalogue # 05040) and incubated at 37 °C and 5% CO_2_ with a medium change every second day. Once cells reached 50–70% confluency, they were dissociated using the Animal Component-Free (ACF) Cell Dissociation Kit (STEMCELL Technologies, catalogue # 05426) following the manufacturer’s instructions.

### Cultureware preparation

All plates used in organoid generation and in downstream assays were coated with Anti-Adherence Rinsing Solution (STEMCELL Technologies, catalogue # 07010). For 24 well plates, 500 μL of Anti-Adherence Rinsing Solution were added to each well to be used and the plate was centrifuged for 10 min at 1300 g. Anti-Adherence Rinsing Solution was then removed, and the well was washed once with 1 mL DMEM/F12 (STEMCELL Technologies, cat # 36254). After removing the wash medium the wells were used directly, or 500 μL fresh DMEM/F12 was added and they were stored for up to one week at 37 °C. For other plate formats, volumes were adjusted accordingly.

### Generation of apical-out airway organoids

Aggregates were generated using PneumaCult Apical-Out Airway Organoid Medium (STEMCELL Technologies, catalogue # 100-0620), following the manufacturer’s instructions. Briefly, hBECs expanded in PneumaCult-Ex Plus Medium were harvested and seeded in 24-well (STEMCELL Technologies, cat # 34411) or 6-well (STEMCELL Technologies, cat # 34421) AggreWell 400 plates at a concentration of 100 cells per microwell unless otherwise stated. For 24-well AggreWell plates, cells were seeded in 1 mL of PneumaCult Apical-Out Airway Organoid Medium, prepared as per the manufacturer's instructions. The AggreWell plate was centrifuged for 3 min at 100 g in order to sediment and aggregate the cells to the bottom of each microwell. Cultures were then incubated for 24 h to 144 h at 37 °C and 5% CO_2_. After the aggregates were generated and sufficiently matured, 1 mL fresh PneumaCult Apical-Out Airway Organoid Medium was added to each well. The aggregates were then resuspended using a P1000 pipette and each well equally distributed to two wells of a 24-well plate treated with Anti-Adherence Rinsing Solution. hBECs were incubated in PneumaCult Apical-Out Airway Organoid Medium for a total of 15 days. A 50% medium change was performed every second day. Media volumes were adapted accordingly for use of 6-well AggreWell plates.

### Inversion of Matrigel-embedded AO

Matrigel embedded AO were generated using PneumaCult Airway Organoid Kit (STEMCELL Technologies, cat # 05060) following the manufacturer’s instructions. Briefly, 2500 hBECs were seeded in a Matrigel (SLS, cat # 356231) dome and cultured for 7 days in PneumaCult Airway Organoid Seeding Medium. Matrigel was then removed incubating organoids at 2–8 °C using Gentle Cell Dissociation Reagent (STEMCELL Technologies, cat # 100-0485) on a shaker for 1 h. The organoids were then washed with DMEM/F12 and cultured in suspension for 3 weeks in PneumaCult Airway Organoid Differentiation Medium.

### Dissociation of apical-out airway organoids and ciliated cell counts

Apical-out airway organoids were harvested on day 15, transferred to a 15 ml tube and centrifuged at 150 g for 5 min. Supernatant was removed, and organoids were washed once with DMEM/F12. Organoids were then resuspended in TrypLE Express (Fisher Scientific, cat # 11558856) and incubated for 5 min at RT, before being dissociated to single cells by pipetting vigorously with a P1000 pipette. The single-cell suspension was diluted 1:1 with Trypan Blue (STEMCELL Technologies, cat # 07050) and was then loaded to a hemocytometer where the cells were counted manually.

### Organoid motility, size and number

Fully mature organoids were imaged using a Leica DMi8 or an EVOS M5000. Percentage of motile organoids was assessed by visually inspecting organoids and manually counting organoids that displayed beating cilia on the outer side. Organoid size was measured by imaging mature organoid cultures and using Fiji^34^ to determine the Feret diameter.

### Propagation of viruses

Enterovirus D68 (EV-D68) 1348 (strain number 4310901348, clade A1) was isolated by the National Institute of Public Health and the Environment (Dr. A. Meijer) and was kindly provided by Prof. Dr. F. J. M. van Kuppeveld (Utrecht University, The Netherlands). Culture strains of Influenza A/ned 177/08 H3 (IAV), Influenza B 20550611 H3 (IBV) and Rhinovirus A16 (RV-A16) were isolated at Amsterdam UMC.

EV-D68 was cultured on RD99 cells maintained in Eagle's Minimum Essential Medium (EMEM; Lonza, cat # 12-611Q) supplemented with 8% Heat-Inactivated Fetal Bovine Serum (HI-FBS; Sigma-Aldrich, cat # F9665-500ML), 100 U-mg/mL Penicillin- Streptomycin Mixture (Lonza BioWhittaker, cat # 09-757F), Non-Essential Amino Acids (NEAA; ScienCell Research Laboratories, cat # 0823) and 200 nM L-Glutamine (Lonza, cat # BE17-605E). IAV, IBV and RV-A16 were cultured on MDCK cells supplemented with 8% HI-FBS (Sigma-Aldrich), 100 U-mg/mL Penicillin–Streptomycin Mixture (Lonza BioWhittaker), NEAA (ScienCell Research Laboratories) and 200 nM L-Glutamine (Lonza). TCID50 of virus stocks and samples was determined using the Reed and Muench method^35^.

### Infection of Ap-O AO and antiviral testing

Terminally differentiated apical-out airway organoids were filtered through a 37 μm reversible strainer (STEMCELL Technologies, cat # 27215) and plated in 400 ul of fresh PneumaCult Apical-Out Airway Organoid Medium without Heparin. EV-D68 was infected at 0.05 Multiplicity Of Infection (MOI) and IAV, IBV and RV-A16 infected at 0.2 MOI. MOI was calculated by using the average number of cells per organoid and assuming each well contained approximately 400 organoids. Infected organoids were incubated for 2 h at 37 °C and 5% CO_2_ and unbound viral particles were subsequently washed by transferring the inoculated organoids to a 15 ml tube, centrifuging at 140 g for 5 min and washing twice in DMEM/F12. The organoid pellet was resuspended in PneumaCult Apical-Out Airway Organoid Medium. Mock-infected organoids were analysed in parallel with infected. For the testing of antivirals effect in EV-D68 replication, 2.5 μM Rupintrivir AG7088 (Sigma, cat # PZ0315) or 5 μM Itraconazole (Santa Cruz, cat # sc-205724) were added to the medium after reconstitution in DMSO (Sigma, cat # D2650). At the designated timepoints, 100 μL of culture medium supernatant was harvested for detection of viral load and titer. 100 μL of fresh medium was provided to the cultures following sampling. The viability of Ap-O AO following infection and treatment was assessed using CellTiter-Glo (Promega, cat # G9681) following the manufacturer’s instructions.

### Quantitative PCR

Total RNA was isolated from Ap-O AO using the Qiagen RNeasy Mini Kit (cat # 74106) as per the manufacturer’s protocol. 500 ng of RNA was DNase treated (Invitrogen, cat # AM2222) as per the manufacturer’s protocol and then reverse-transcribed to cDNA using SuperScript III (Invitrogen, cat # 18080-044). TaqMan gene-specific assay primers and probes were obtained from Integrated DNA Technologies (Table 1) and reaction mixture from Applied Biosystems (cat # 10575385). Samples were amplified as follows: denaturation at 95 °C for 20 s followed by 50 cycles at 95 °C for 1 s and 60 °C for 20 s. The mRNA expression levels of cellular genes were normalized with that of *TBP*, as it showed to have the smallest standard deviation amongst a panel of reference genes.

**Table 1:** Accession number of assays used to characterize apical-out airway organoids.

Gene	IDT assay
*KRT5*	Hs.PT.58.14446018
*ITGA6*	Hs.PT.58.453862
*FOXJ1*	Hs.PT.58.40371261
*TUBB4B*	Hs.PT.58.15334509.g
*SCGB1A1*	Hs.PT.58.1190800
*MUC5AC*	Hs.PT.58.25491641.g
*KRT18*	Hs.PT.58.4200217
*TSLP*	Hs.PT.58.22464960
*FOXI1*	Hs.PT.58.40514934
*TBP*	Hs.PT.39a.22214825

To quantify virus replication, 25 μL of the supernatant sample was lysed and RNA was extracted using the ISOLATE II RNA Mini Kit (BIOLINE, cat # BIO-52073), as per manufacturer’s instructions. Viral RNA was eluted in 60 μl elution buffer and cDNA was synthesized using SuperScript III (Invitrogen) with random Hexamer primers**.** qPCR was performed in a CFX96 Real-Time PCR Detection System (Biorad) with LightCycler TaqMan Master Mix (Roche, cat # 04735536001) using virus-specific primers and probes (Table 2) to detect viral gene copy numbers. Viral gene copy number was determined by absolute quantification using a plasmid expressing a conserved region of the gene. In all tested conditions, the levels of viral RNA in mock-infected organoids were below the detection limit.

**Table 2:** Sequences of primers and probes used to detect viral RNA load.

Target	Primer/probe	Sequence 5′ to 3′	Label
RV-A16	Forward	AGS CTG CGT GGC KGC C	
	Reverse	ACA CGG ACA CCC AAA GTA GT	
	Probe	TCC TCC GGC CCC TGA ATG YGG CTA AYC	6FAM-BBQ
IBV	Forward	TCG CTG TTT GGA GAC ACA AT	
	Reverse	TTC TTT CCC ACC GAA CCA	
	Probe	AGA AGA TGG AGA AGG CAA AGC AGA ACT	6FAM-BBQ
IAV	Forward	GAC AAG ACC AAT CCT GTC ACY TCT G	
	Reverse	AAG CGT CTA CGC TGC AGT CC	
	P610	TTC ACG CTC ACC GTG CCC AGT GAG C	6FAM-BBQ
EV-D68	Forward	TGT TCC CAC GGT TGA AAA CAA	
	Reverse	TGT CTA GCG TCT CAT GGT TTT CAC	
	Probe1	ACC GCT ATA GTA CTT CG	6FAM-BBQ
	Probe2	TCC GCT ATA GTA CTT CG	6FAM-BBQ

### Immunofluorescence staining

Apical-out organoids were fixed in Dents fixative (20% DMSO, 80% methanol) before being permeabilized with 1% Triton X-100 (Sigma Aldrich, cat # 10789704001) in PBS. Permeabilized organoids were blocked with 5% Normal Goat serum in PBS + 0.1% Tween-20 (Sigma Aldrich, cat # P9416) + 0.2% Triton X-100 (PBSTT). Primary antibodies were diluted in PBSTT and incubated with the cells for 3 days or overnight at room temperature in a tube with gentle agitation. Ap-O AO were stained for the epithelial markers acetylated α-TUBULIN (Sigma, cat # T7451), KERATIN 5 (Biolegend, cat # 905501), ZO-1 (Thermo Fisher Scientific, cat # 339188) and MUC5AC (Abcam, cat # ab212636). Goat anti-Mouse IgG2b Cross-Adsorbed Secondary Antibody, Alexa Fluor 568 (Thermo Fisher Scientific, cat # A-21144), Goat anti-Mouse IgG1 Cross-Adsorbed Secondary Antibody, Alexa Fluor 488 (Thermo Fisher Scientific, cat # A-21121) and Donkey anti-Rabbit IgG (H + L), Alexa Fluor 647 (Thermo Fisher Scientific, cat # A-31573) were used respectively for the primary antibodies. Cells were washed with PBSTT and the respective secondary antibody was incubated at room temperature in a tube with gentle agitation for 3 days or 1 h. Longer incubation times were used to ensure appropriate level of interaction between the antibodies and their respective epitopes and improve image clarity. Cells were washed again with PBSTT before staining with 4′, 6-diamidino-2-phenylindole (DAPI, Cayman Chemical, cat # 14285) and imaged with LEICA SP8. To visualize viral particles in infected organoids, Ap-O AO were harvested directly after infection (0 h post infection, hpi) and 6 hpi, and subjected to the procedure described above for the EV-D68 viral protein VP1 (Genetrex, cat # GTX132313) and dsRNA (SCICONS, cat # 10010200). Non-infected Ap-O AO were processed in parallel as negative controls. Incubation with secondary antibodies only was performed to validate the absence of non-specific antibody binding in all tested conditions. To determine the number of nuclei per organoid, 60 Ap-O AO were processed as above and stained with DAPI. DAPI-positive cells were manually counted using a LEICA SP8.

### Statistical analysis

For each biological replicate, infections were done in triplicate, and *n* represents the number of biological replicates. Data distribution was assessed with the Shapiro–Wilk test. Data sets with normal distribution were analysed with Students’ T-test. Data sets with non-normal distribution were analysed using Mann–Whitney test. All tests were performed using GraphPad Prism (version 8.1.1 for Windows GraphPad Software, San Diego, California USA) with significance set at *p* < 0.05. Graphs were generated using either GraphPad Prism (version 8.1.1 for Windows GraphPad Software, San Diego, California USA) or Project Jupyter^36^.

## Supplementary Information

Supplementary Information 1.

Supplementary Video 1.
