# Supplementary material for: Apical-out airway organoids as a platform for studying viral infections and screening for antiviral drugs
Source: Sci Rep. 2022 May 10;12:7673. doi: 10.1038/s41598-022-11700-z (PMC9089294; doi:10.1038/s41598-022-11700-z)
Supplement: Supplementary file 1 — Supplementary Information 1. [file 41598_2022_11700_MOESM1_ESM.pdf]

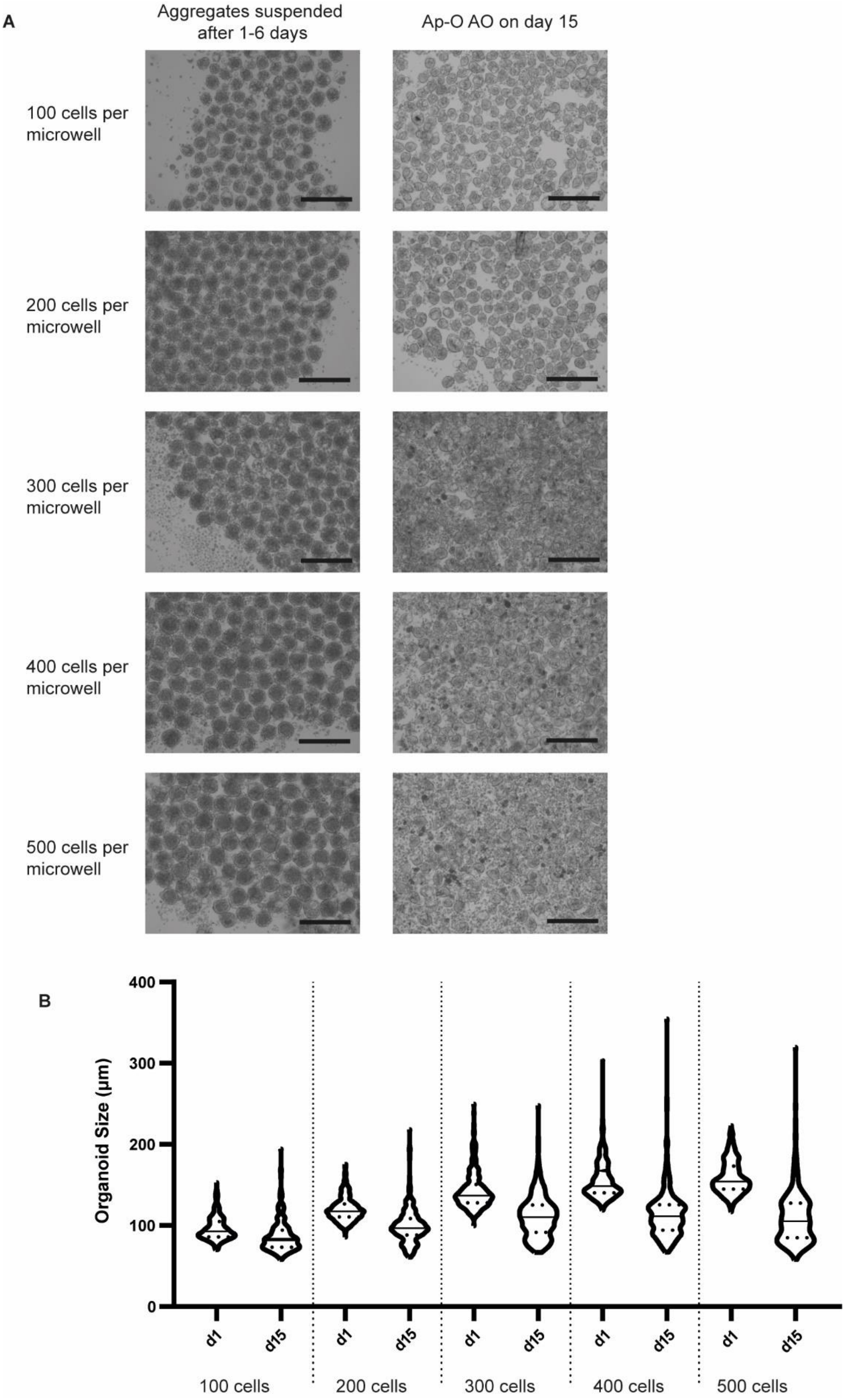

Supplementary Figure 1: **Optimization of the microwell seeding density**

A. Brightfield images of different cell densities seeded in AggreWell directly after suspension (day 1-6) and at the end of the differentiation protocol (day 15). Cell shedding can be observed from larger aggregates (scale bars = 300 µm).
B. Violin plots of the Feret diameters from aggregates after transfer to suspension culture and Ap-O AO at day 15 using different initial seeding densities per microwell. Doted lines represent the quartiles, straight lines represent the median. Exactly 100 Ap-O AO were measured per donor for each condition (n = 3).

Supplementary Video 1: **Apical-out Airway organoids display visible beating cilia on the outer side.**

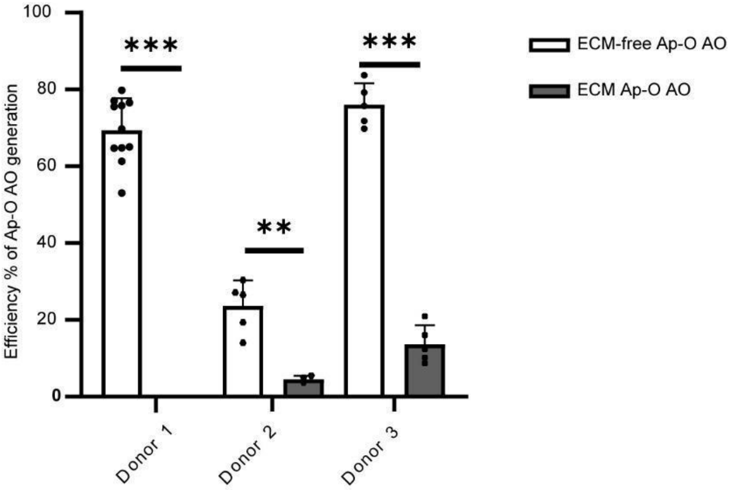

Supplementary Figure 2: **Comparison of the apical out airway organoid generation efficiency**

Ap-O AO forming efficiency following aggregate generation and suspension culture of ECM-embedded organoids. Samples labelled ECM-Free Ap-O AO indicate apical-out organoids generated with our ECM-free method; samples labelled ECM Ap-O AO indicate apical-out organoids generated using the method published by Salahudeen *et al*[13]. Efficiency was calculated as the number of aggregates or organoids prior to initiation of the suspension culture divided by the number of formed Ap-O AO at end point. Points represent technical replicates. Data are presented as mean ± SD of at least 5 independent technical replicates. P value is calculated by unpaired Students' t-test. Single asterisks indicate p<0.05, double asterisks p<0.01 and three asterisks p<0.001.

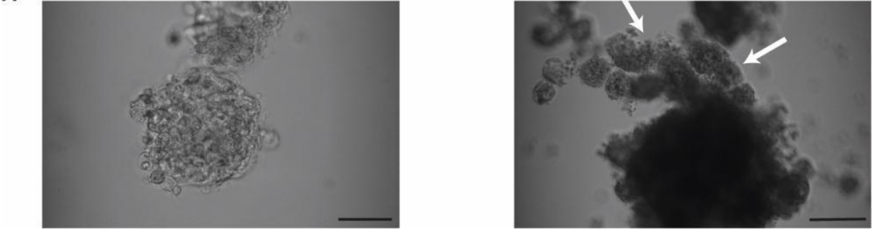

Supplementary Figure 3: **Representative images of ECM-embedded organoids after ECM removal and suspension culture**

A. Representative brightfield image of a deteriorating ECM-derived organoid in suspension culture (scale bar = 50 µm).
B. Brightfield image showing a large airway organoid derived from organoid-organoid fusion. White arrows indicate organoids that are fused but not completely merged (scale bar = 200 µm).

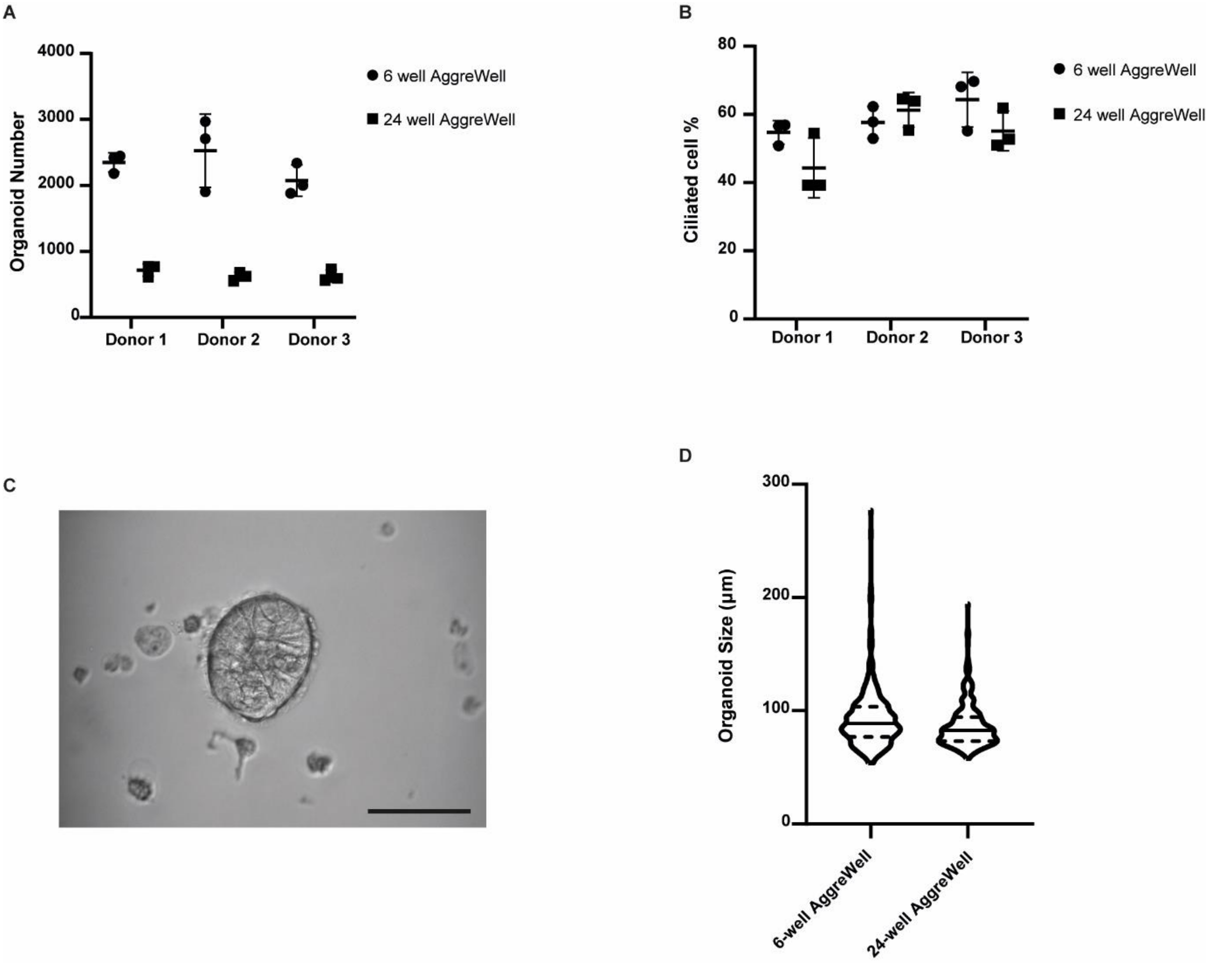

Supplementary Figure 4: **Comparison of Ap-O AO generated from 24 and 6 well AggreWell plates**

A. Number of generated organoids per well of 6 and 24-well AggreWell plate across three different donors. Data points represent independent wells (n = 3).
B. Ciliated cell percentage of generated Ap-O AO at day 15 from 6 and 24-well AggreWell plates. Data points represent measurements taken from independent wells of a 24-well plate (n = 3).
C. Representative brightfield image of terminally differentiated Ap-O AO at day 15 from 6-well Aggrewell plate (scale bar 75 µm).
D. Violin plots depicting the frequency of different Feret diameters in p5 Ap-O AO from 3 donors generated in 6 or 24-well AggreWell plates. Doted lines represent the quartiles, straight lines represent the median. 100 organoids were equally counted from 3 wells for each donor and plate format.

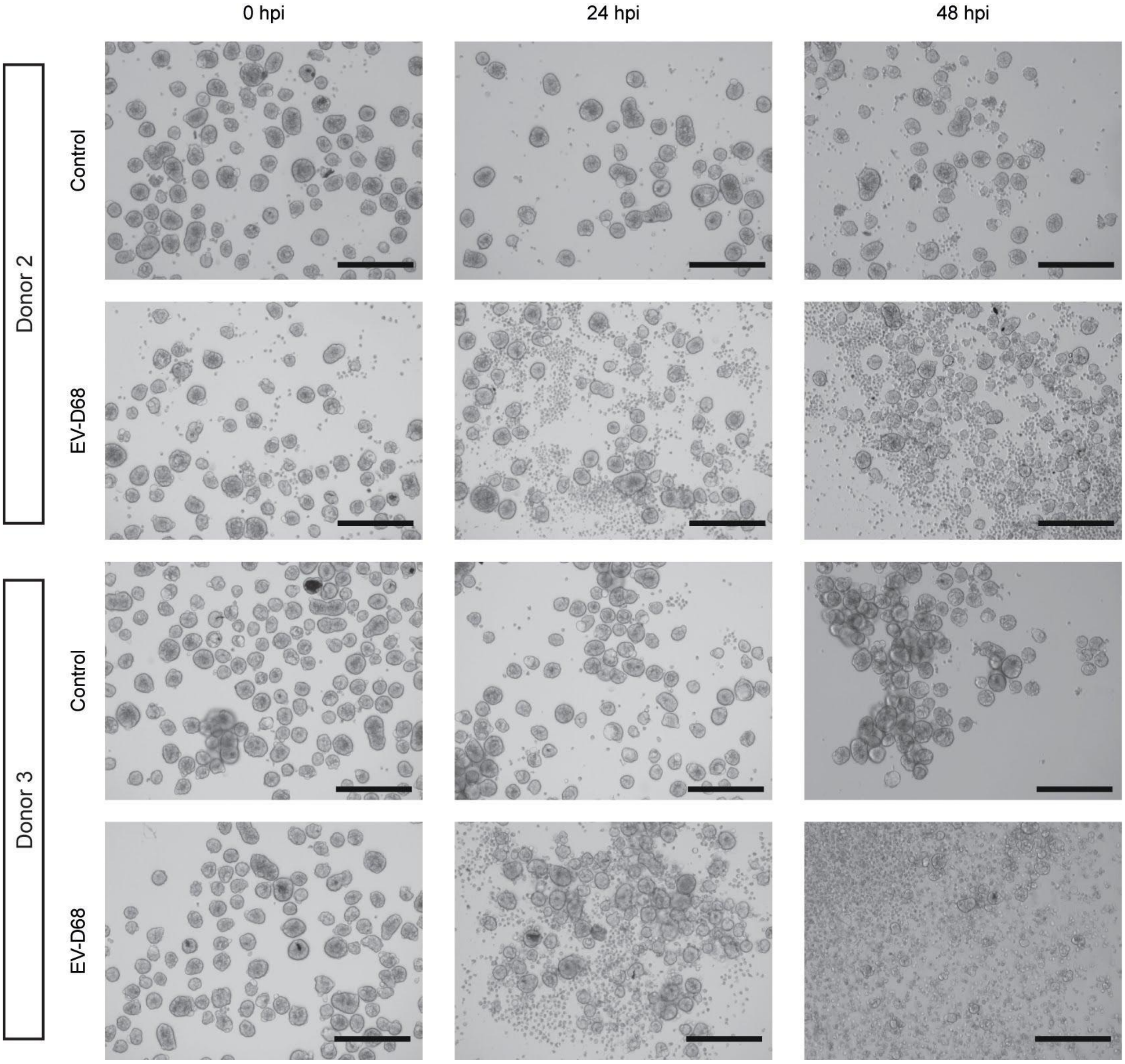

Supplementary Figure 5: **CPE observed in donors 2 and 3.**

Representative brightfield images showing the cytopathologic effect of EV-D68 infection over 48 hours in the remaining 2 donors (scale bars = 300 µm).

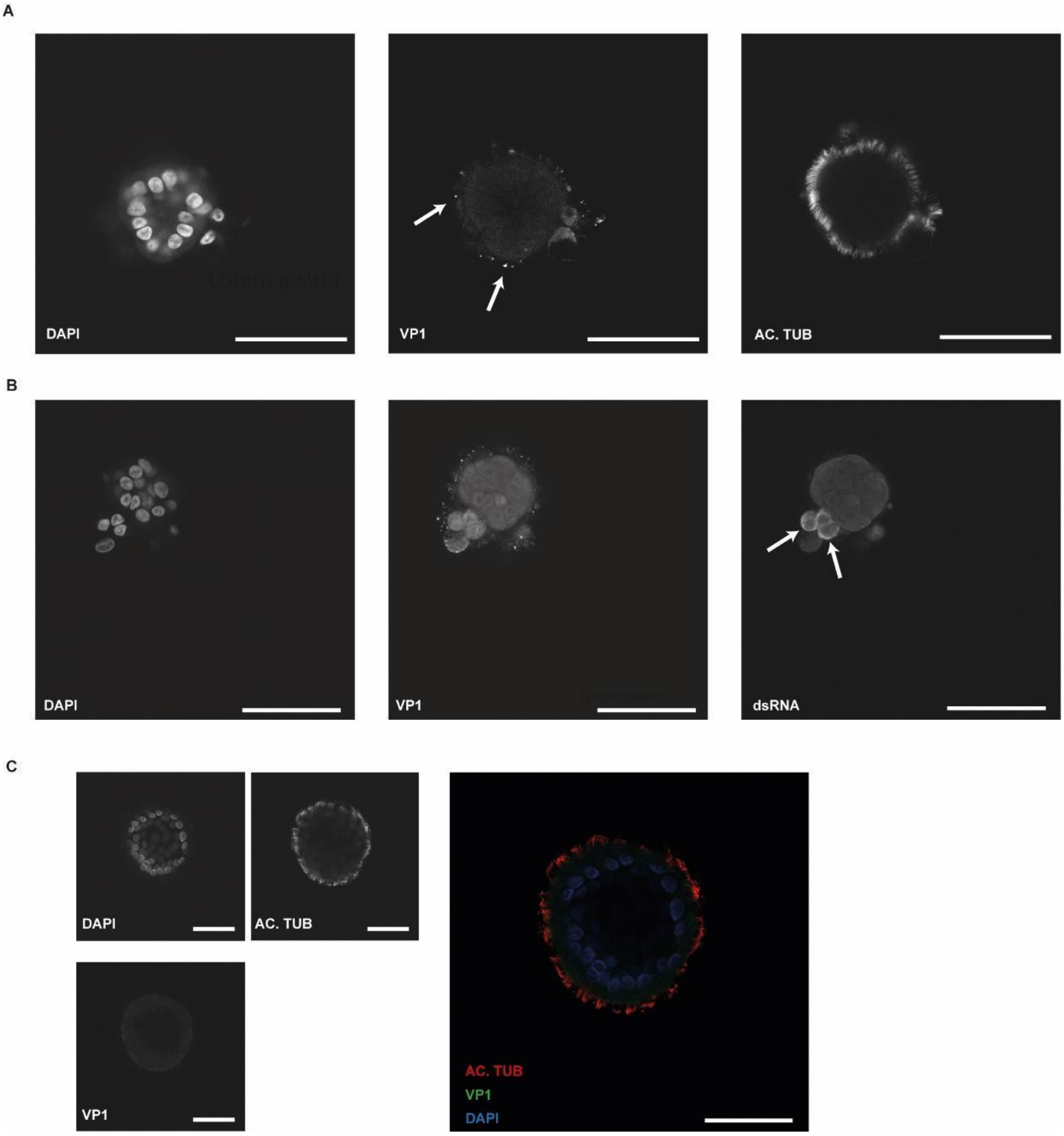

Supplementary Figure 6: **ICC images of Ap-O AO infected with EV-D68**

A. Single-channel images showing the distribution of DAPI, VP1 and AC. TUB in Ap-O AO after 0 hpi with EV-D68. Arrows point at bound viral particles (scale bars = 50 µm).
B. Single-channel images showing the distribution of DAPI, VP1 and dsRNA from Ap-O AO after 6 hpi with EV-D68. Arrows point at infected cells with ongoing replication (scale bars = 50 µm).
C. Single-channel and merged images showing the distribution of DAPI, VP1 and AC. TUB in non-infected Ap-O AO (scale bars = 50 µm).

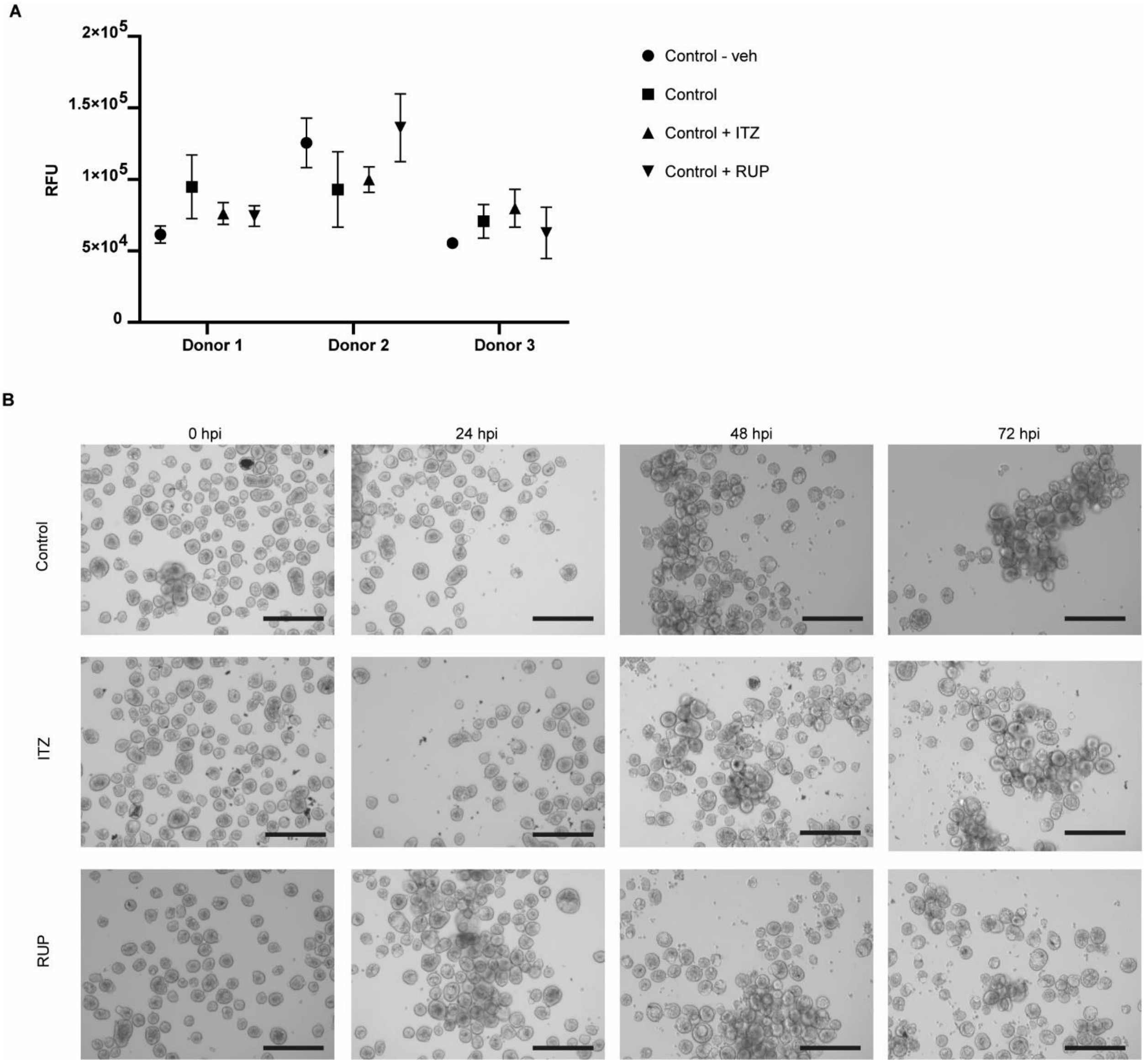

Supplementary Figure 7: **Effect of ITZ and RUP in Ap-O AO**

A. Relative Fluorescent Units (RFU) indicating changes in viability of Ap-O AO following addition of ITZ or RUP for 72 hours. Single points represent technical replicates. Bars depict the mean with SEM (n = 3).

B. Morphology of Ap-O AO following addition of Itraconazole or Rupintrivir. Morphological changes could not be observed following addition of the antivirals (scale bars = 300 µm).
